# Fatal poisonings in Northern Finland: causes, incidence, and rural-urban differences

**DOI:** 10.1186/s13049-017-0431-8

**Published:** 2017-09-08

**Authors:** Lauri Koskela, Lasse Raatiniemi, Håkon Kvåle Bakke, Tero Ala-Kokko, Janne Liisanantti

**Affiliations:** 1 Oulu University Hospital, Department of Anesthesiology, Division of Intensive Care Medicine, P.O.BOX 21, 90029 OYS Oulu, Finland; 2 Oulu University, Medical Research Center, Study Group of Surgery, Anesthesiology and Intensive Care, Oulu, Finland; 3 Centre for Pre-Hospital Emergency Care, Oulu University Hospital, Oulu, Finland; 4 Anaesthesia and Critical Care Research Group, University of Tromsø, Tromsø, Norway; 5 Mo i Rana Hospital, Helgeland Hospital Trust, Mo i Rana, Norway; 6 Department of Anaesthesiology and Intensive Care, University Hospital of North Norway, Tromsø, Norway

**Keywords:** Poisoning, Intoxication, Alcohol, Finland, Mixed poisoning, Rurality, Suicide

## Abstract

**Background:**

In this study we evaluate differences between rural and urban areas in the causes and incidence of fatal poisonings.

**Methods:**

Data from all fatal poisonings that occurred in Northern Finland from 2007 to 2011 were retrieved from Cause of Death Registry death certificates provided by Statistics Finland. The demographics and causes of fatalities were compared between rural and urban areas. Incidences were calculated based on the population data.

**Results:**

There were a total of 684 fatal poisonings during the study period and 57.9% (*n* = 396) occurred in the urban population. Ethanol was the most common primary poisoning agent in cases of fatal poisoning, accounting for 47.5% of cases in urban areas and 68.1% in rural areas (*P* < 0.001). Fatal poisonings caused by psychoactive pharmaceutical products and opioids were more common in urban areas (28.3% compared to 18.0%, *P* < 0.001).

The crude incidence of fatal poisonings in the study area was 18.8 (17.4–20.2) per 100,000 inhabitants per year and there was no difference in incidence between urban and rural areas. In the youngest age group (15 to 24 years), the incidence of fatal poisonings observed in urban areas was two times higher than that in rural areas.

**Discussion:**

Higher rate of fatal ethanol poisonings in rural areas could be linked to higher alcohol consumption in rural areas and also differences in drinking behaviour. Higher incidence of poisoning suicides in urban areas could be due to availability of different toxic agents as a suicidal method. Preventive measures could be key in reducing the number of fatal poisonings in both areas, as most of the fatal poisonings still occur outside hospital.

**Conclusion:**

There was a higher rate of fatal ethanol poisoning in rural areas and higher rate of fatal poisoning related to psychoactive pharmaceutical products and opioids in urban areas. There were twice as many fatal poisonings in the youngest age group (15–24 years) in urban areas compared to rural areas, and suicide was more common in urban areas.

## Background

Causes of poisoning can vary depending on urbanization rate. For example, there is a higher rate of fatal poisoning due to pesticides in rural areas in South-East Asia and Africa, and reports of other differences in types of poisoning between rural and urban contexts can be found in the literature [1, 2]. In the United States, the urbanization rate has been shown to have an effect on drug poisoning deaths, and while heroin overdoses have been increasing in rural areas, they are still more prevalent in urban areas [3, 4]. An Irish study found a higher mortality rate for poisoning in urban areas over the years 1980–2000 [5]. However, there are few studies aside from these articles comparing poisonings between rural and urban areas that can also be generalized to the Nordic countries or to Europe. Studies focusing on geographical differences in poisonings could be used to target preventative interventions to the at-risk population, as well as to organize education and access to emergency medical services.

A recent study from Northern Finland indicated that there are regional differences between rural and urban areas in the use of health care services and in well-being [6]. However, while poisoning related mortality is a significant problem in Finland, regional differences in poisonings have not been studied [7, 8].

The aim of this study is to investigate and compare the incidence and characteristics of fatal poisoning between urban and rural areas in northern Finland using autopsy reports that include full and detailed toxicological screening.

## Methods

### Study population

This is a retrospective study comparing an urban and a rural cohort. We identified all deaths from poisoning that occurred within the study areas between January 1, 2007, and December 31, 2011 from the Finnish Cause of Death Registry. The Finnish Cause of Death Registry is a national archive in which the deaths in the Finnish population from 1936 to 1965 were registered. Deaths in subsequent years were registered by Statistics Finland. Statistics Finland allows the data from death certificates to be used for research purposes with permission and after evaluation of the protocol. We defined the fatality as a poisoning if the cause of death was registered as International Classification of Disease (ICD-10) diagnosis code X40–49, X60–69, X85–90, Y10-Y19, T36-T50, T51-T65, Y34, Y57, or Y84 [9]. Only victims residing in Finland were included in the study, and fatal poisoning linked to medical care and poisoning deaths related to breathing combustible gas were excluded. Death certificates based on medicolegal post-mortem examination and toxicological screening as well as medical records were reviewed for each victim. Finnish law states that medicolegal autopsy should be performed if the cause of death is or is suspected to be crime, accident, suicide, poisoning, or is unknown [10].

### Toxicology

The cause of the poisoning was determined by post-mortem screening for alcohol, pharmaceutical products, and illicit drugs conducted at the Hjelt Institute, University of Helsinki. Biopsies are taken from the deceased for toxicological screening, and the concentrations of each potentially poisonous chemical found in the sample are compared to their physiological or therapeutic concentrations by the forensic pathologist, who also considers the specific pharmacological properties of the relevant molecules. These screening results are combined with police reports from the scene, observations from the external inspection of the deceased, autopsy findings, and results from histological or other analyses [11].

### Variables studied

Our dataset consists of patient demographics, place of death, intention of intoxication (defined as accident, self-inflicted, or undetermined), main causative toxic agent, and alcohol involvement. The intention of the poisoning incident was assigned based on the category listed on the death certificate. Suicidal intent requires physical evidence of that intent, such as a suicide letter. Death was defined as ‘pre-hospital’ if the death occurred before hospital admission, and the death was defined as ‘death after ambulance arrival’ if the victim had signs of life after the ambulance crew arrived. If more than one significant agent was detected in the screening the cause of the fatal poisoning was classified as “multiple ingestion”. We defined “illicit street drugs” as substances that are not used by health care professionals, therefore pharmaceuticals such as benzodiazepines or opioids aren’t defined as illicit drugs, even if the deceased obtained them from an illegal source. In this study we classified opioids based on their comparative strength: codeine and tramadol as weak opioids, buprenorphine as a medium opioid and oxycodone, fentanyl, methadone, propoxyphene and remifentanil as strong opioids.

### Study area and definition of rurality

In this study, rurality was determined by municipality data. In Finland municipalities are the smallest self-governing local units with their own population and region. The study area consisted of 72 municipalities in Northern Finland with a population of 728,847 inhabitants in 2011 and which encompasses the five northernmost hospital districts in Finland. The driving distance from the northernmost municipality to the nearest central hospital is approximately 5 h. In this area, 317,634 (44%) inhabitants resided in rural municipalities and 411,213 (56%) resided in urban municipalities in the chosen index year of 2011. The municipalities in the study area were defined as rural or urban according to the Statistics Finland grouping of municipalities [12]. However, while Statistics Finland groups municipalities into three categories, rural, semi-urban, and urban, in this study municipalities were divided into only two groups, urban and rural, and the latter also included semi-urban municipalities. Statistics Finland defines urban municipalities as those municipalities in which at least 90% of the population lives in urban settlements or in which the population of the largest urban settlement is at least 15,000 [13]. Average people density in urban municipalities of the study area was 72.85 people per square kilometre and in rural municipalities 5.26 people per square kilometre [14]. Deaths were allocated to the urban or rural group according to the municipality of residence at the time of the fatal poisoning event.

### Statistical analysis

Statistical analysis was performed using IBM SPSS Statistics 24 software (IBM SPSS Statistics for Windows, Version 24.0, Armonk, NY, USA). Summary calculations are presented as median values with 25th and 75th percentiles (25-75th percentiles) and were analysed using Mann-Whitney’s non-parametric test. Categorical variables were analysed using Pearson’s χ2 test and Fisher’s exact test if needed. Incidence rates were calculated according to age groups with ten year intervals. Two-tailed *P* values less than 0.05 were considered statistically significant.

### Ethics

The study protocol was approved by the Oulu University Hospital administration (Reference no. 110 12,015), the Regional Ethics Committee (Reference no. 982013), and Statistics Finland (Reference no. TK53–1151-13).

## Results

There were a total of 684 fatal poisonings during the five year study period. 57.9% (*n* = 396) of the deaths occurred in the urban population. Medicolegal autopsy was performed for all (*n* = 684) victims.

The majority of the deaths were pre-hospital (92.8%). 76.9% of the victims were found dead on scene and the location of the poisoning was the home of the victim or an acquaintance in 90.1% of cases.

The victims in urban areas were younger (47.5 years compared to 52 years, *P* < 0.001) and the deaths were more often with suicidal intent (82 of 396 (20.7%) compared to 40 of 288 (13.9%, *P* = 0.02)), (Table 1). Ninety-five of 396 (24.0%) deaths in urban areas occurred in a population less than 35 years of age, in contrast to 30 of 288 (6.9%, *P* < 0.001) in rural areas. Overall the most common fatal poisoning intent was accident (519 (75.9%)), followed by suicide (122 (17.8%)) and undetermined intent (43 (6.3%)).

**Table 1:** Demographics of the 684 poisoning deaths

	Urban *n* = 396	Rural *n* = 288	*P*
Age	47.5 (36–57)	52 (44–59)	<0.001
Gender (m/f)	292 / 104	226 / 62	0.090
Suicidal intention	82 (20.7)	40 (13.9)	0.022
Found dead on scene	313 (79.0)	213 (74.0)	0.254
Death before ambulance arrival (signs of life may have been observed before ambulance arrival)	46 (11.6)	42 (14.6)	
Death after ambulance arrival (Signs of life upon arrival of ambulance crew)	13 (3.3)	8 (2.8)	
Death during transport	1 (0.3)	4 (1.4)	
Death during hospital stay	23 (5.8)	21 (7.3)	
Location of poisoning			0.087
Home or acquaintance	349 (88.1)	267 (92.7)	
Settlement or city area	16 (4.0)	6 (2.1)	
Outdoors	15 (3.8)	11 (3.8)	
Health facility	7 (1.8)	3 (1.0)	
Prison	9 (2.3)	1 (0.3)	

Data are presented as percentages and medians (25th–75th percentiles)

### Causes of the fatal poisonings

**Fig. 1:**
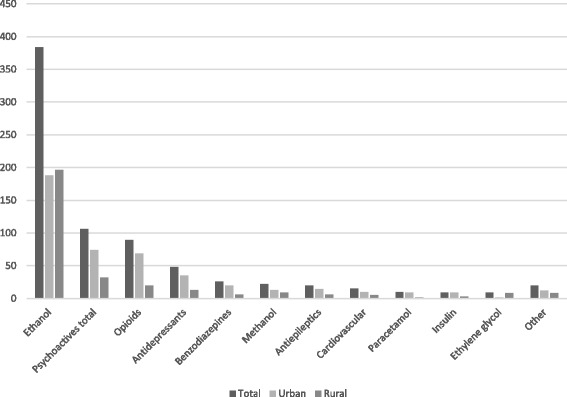
Number of primary poisoning agents in fatal poisonings in Northern Finland 2007–2011

Ethanol poisonings accounted for 384 of 684 (55.7%) of fatal poisonings, and were significantly higher in rural areas (Table 2). Multiple ingestions were more frequently found in urban areas compared to rural areas (233 of 396 (58.8%) compared to 118 of 288 (41.0%, *P* < 0.001)). Ethanol was involved as the main toxic agent or found in multiple ingestions in 231 of 228 (80.2%) of fatal poisonings in rural areas and in 270 of 396 (68.2%) in urban areas (*P* < 0.001). Psychoactive pharmaceutical products and opioids were more often found in urban fatal poisonings (Fig. 1). Illicit street drugs were not found to be primary poisoning agents in any of the fatalities.

**Table 2:** The primary poisoning agents of the 684 fatal poisonings

	Total *n* = 684	Urban *n* = 396	Rural *n* = 288	*P*-Value
Ethanol	384 (55.7)	188 (47.5)	196 (68.1)	<0.001
Methanol	22 (3.2)	13 (3.3)	9 (3.1)	>0.9
Ethylene glycol	9 (1.3)	1 (0.3)	8 (2.8)	0.005*
Psychoactives	106 (15.4)	74 (18.7)	32 (11.1)	0.007
Neuroleptics	32	19	13	
Benzodiazepine	26	20	6	
Antidepressant	48	35	13	
Antiepileptics	20 (2.9)	14 (3.5)	6 (2.1)	0.27
Opioids	89 (12.9)	69 (17.4)	20 (6.9)	<0.001
Weak	58	43	15	
Medium	12	11	1	
Strong	19	15	4	
Paracetamol	10 (1.5)	9 (2.3)	1 (0.3)	0.03*
Insulin	9 (1.3)	6 (1.5)	3 (1.0)	0.43*
Cardiovascular	15 (2.2)	10 (2.5)	5 (1.7)	0.49
Beta blockers	8	6	2	
Ca2+ channel blockers	3	3	0	
Other cardiovascular drugs	4	1	3	
Other	20 (2.9)	12 (3.0)	8 (2.8)	0.84

*P*-value calculated using Fisher’s exact*

### Incidences of fatal poisoning

**Fig. 2:**
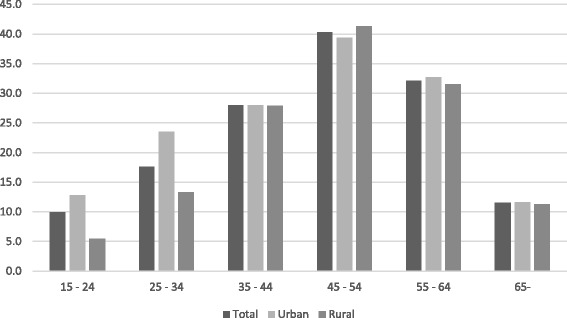
Incidence of fatal poisonings in rural and urban areas in different age groups. (Incidence / 100,000 / per year)

The crude incidence of fatal poisoning in the study area was 18.8 (17.4–20.2) per 100,000 inhabitants per year, and there were no differences in incidence between urban and rural areas. The highest incidence was found in the 45 to 54 year age group (Fig. 2). In the youngest age group (15 to 24 years) the incidence of fatal poisoning was two times higher in urban areas (Fig. 2). The crude incidence of fatal poisoning by substances other than ethanol was 5.8 (4.7–7.1) for rural areas, in contrast to 10.1 (8.8–11.6) for urban areas.

## Discussion

The main finding of this study was that the causes of fatal poisoning differ significantly between rural and urban areas, while overall incidence does not. Rural areas had a higher rate of fatal ethanol poisoning, while urban areas had a higher rate of fatal poisoning related to psychoactive pharmaceutical products and opioids. We also found that the incidence of fatal poisoning in the youngest age group (15–24 years of age) was twice as high in urban areas compared to rural areas. Finally, poisoning with suicidal intent was more common in urban areas.

### Causes of fatal poisonings

Though the overall mortality rate from poisoning was not significantly different between rural and urban areas, we did find significant differences in the agents ingested between the two areas. The most common toxic agent was ethanol, which was also involved in the majority of multiple ingestions. Alcohol abuse is a substantial health problem in Finland, and alcohol-related deaths in the working population accounted for 14.5% of total mortality in this age group in 2014 [15]. We found a higher rate of fatal ethanol poisonings in rural areas. In contrast to the fatal ethanol poisoning majority in rural areas, non-ethanol poisoning accounted for more than 50% of the fatal poisonings in urban areas. Notably, fatal poisoning due to psychoactive pharmaceutical products and opioids were significantly higher in urban areas, and we note that the rate of fatal opioid poisoning was more than double in urban areas (Table 2). There may be cultural and behavioural differences between areas based on urbanization rate, and in Finland, alcohol consumption is generally higher in rural areas relative to urban areas, which could partially explain this difference [16]. People in urban areas may also have easier access to pharmaceutical products, and therefore run a higher risk of pharmaceutical intoxication. Relatedly, urban areas in Finland are reported to have a higher use of anti-depressants [16], and poisoning deaths with pharmaceutical products (especially prescription opioids) have been shown to be associated with mental health problems [17–19]. We were not able to retrieve data related to drug abuse by the deceased, but a nationwide study showed that 26% of fatal opioid poisonings during 2010–2011 were associated with drug abuse [20].

### The incidence of fatal poisoning

The incidence of fatal poisoning in our study was relatively high compared to other Nordic countries, as demonstrated previously [21], but significant difference in crude incidence between rural and urban areas was not found.

The overall incidence of fatal poisoning in the present study was comparable to a previous Finnish study [7], and the incidence of fatal poisoning due to pharmaceutical agents in southern Sweden was 6.5/100,000 per year, comparable to the present results [22]. Though the main toxic agents varied between urban and rural areas, the overall incidence of fatal poisonings did not differ. This finding is in contrast to studies outside of Europe, which have shown regional differences in the incidence of poisoning deaths [1, 3]. However, those studies focused primarily on illicit street drugs and pesticides, unlike this study.

**Fig. 3:**
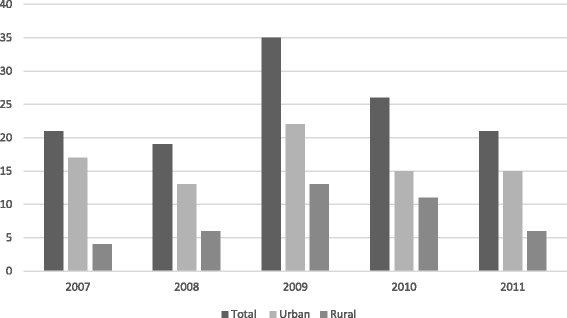
Number of poisoning suicides each year in Northern Finland 2007–2011

When we stratified incidence by age, we found that the incidence of fatal poisonings was higher in urban areas for the 15 to 24 year age group. There could be several reasons for this difference in incidence. We found a higher incidence of suicides in the urban cohort, in contrast to several other studies reporting that suicide is more frequent in rural areas [23–25]. In Finland, suicide is a major cause of death for young people. In 2015, suicide was the cause of death for one in three people aged 20 to 29 and one in six for people aged 35 to 44. One in ten of all suicides was committed by a person under the age of 25, and one in five suicide victims was over 65 years of age [26]. According to Eurostat’s statistics, in 2013 the suicide mortality in Finland for the population under age 65 was approximately 1.5 times higher than the EU average [27]. We demonstrated the trend of fatal poisonings with suicidal intention in our study population in a figure. (Figure 3) As we only investigated poisonings, our findings may be the result of a difference in suicide method between rural and urban areas in younger age groups. Method-specific suicide rates have been demonstrated to be associated with the availability of the method [28], and such differences have also been reported in Finland [29]. In Northern Finland, the crude incidence of suicides resulting from mechanisms other than poisoning has been reported to be slightly lower in urban areas, indicating higher rates of more violent methods of suicides in those areas [30]. This may be a result of the easier access to pharmaceutical products in urban areas and easier access to other possible methods, such as weapons, in rural areas. On the other hand, the higher rates of alcohol consumption seen in rural areas [16] could be another explanation for the difference in incidence in younger age groups, as binge drinking is reported to be associated with lower rates of drug poisoning deaths [3].

### The rate of pre-hospital deaths

Previously, we showed that most fatal poisonings in the same area occur outside the hospital [8]. Earlier access to pre-hospital advanced life support therefore seems to be of little importance when trying to reduce the incidence of fatal poisoning. The absence of a difference in the proportion of pre-hospital deaths in this study suggests that differing access to EMS-services between urban and rural areas does not have a substantial impact on poisoning deaths. It is conceivable that reported differences in urban and rural poisonings are more related to socioeconomic factors and is therefore is not seen in Finland, where there is relative socioeconomic equality [31].

### Limitations

The main limitation of this registry study is that we were not able to link any data with previous medical, social, and economic history. Therefore, we were not able to identify specific risk factors for people who were poisoned with suicidal intent in urban or rural areas. There is also no data on alcohol consumption or psychiatric well-being. We were also not able to consider the concentrations of the toxic agents, so it was not possible to make comparisons such as ethanol poisoning severity. We also note that a high number of the screenings are taken from post-mortem samples, limiting the reliability of comparisons between deceased individuals. Since the study is based on death certificates and most of the deaths occurred outside the health care system, we are also not able to give fully reliable data concerning the motivation of the intake. Suicidal intention could be underestimated, since deaths are not categorized as suicide unless there is clear clinical evidence of that intent.

### Clinical significance

There is little previous research on rural-urban differences in fatal poisonings in Northern Europe. Here we present results based on data covering all poisoning deaths in the study area during the study period with a 100% autopsy rate. The findings indicate that the most efficient way to reduce the rate of fatal poisonings in Northern Finland is to take preventive measures against the abuse of drugs and alcohol. The present study did not find a substantial proportion of preventable deaths occurring in pre-hospital, emergency, or critical care, and this study did not aim to evaluate the benefits of pre-hospital care for the cases severe poisonings in which the victim survived.

It has been shown that alcohol use often reflects parental modeling [32]. It is also necessary to recognize the need for the treatment of depression and other psychiatric disorders in order to prevent suicidal behavior. Specific improvements to emergency and intensive care play a minor role in efforts to decrease poisoning deaths, since only a minority of the deaths occur after contact with health care providers. Reducing longer pre-hospital responses and transportation times for the rural population will not result in significantly lower number of pre-hospital deaths. The impact of brief intervention and referral to the health care system by emergency medical services (EMS) for patients identified to have drug and alcohol problems or suicidal behavior should be studied. The effects of brief interventions on alcohol use have previously been shown to be moderate [33] and beneficial in suicide prevention [34]. In Finland, a large proportion of the patients encountered by EMS are not transported to the hospital [35]. In this setting EMS personnel may have an important role in recognizing alcohol, drug and mental health problems. To educate EMS personnel in recognizing these problems and conducting a brief intervention could be a rational approach.

## Conclusion

There is a higher rate of fatal ethanol poisonings in rural areas, a higher rate of fatal poisonings related to psychoactive drugs and opioids in urban areas, and a two-fold increase in the incidence of fatal poisonings in the youngest age group (15–24 years of age) in urban areas. There were no differences in crude overall incidence of fatal poisonings between rural and urban areas in Northern Finland.

## Abbreviations

EMS: Emergency medical services
